# Mechanism and Protection of Radiotherapy Induced Sensorineural Hearing Loss for Head and Neck Cancer

**DOI:** 10.1155/2021/3548706

**Published:** 2021-12-21

**Authors:** Wenxia Shi, Xue Hou, Xueying Bao, Wei Hou, Xuehua Jiang, Lixin Ma, Xin Jiang, Lihua Dong

**Affiliations:** ^1^Department of Radiation Oncology & Therapy, The First Hospital of Jilin University, Changchun 130021, China; ^2^Jilin Provincial Key Laboratory of Radiation Oncology & Therapy, The First Hospital of Jilin University, Changchun 130021, China; ^3^NHC Key Laboratory of Radiobiology, School of Public Health, Jilin University, Changchun 130021, China; ^4^Ophthalmology Department, The 3rd Affiliated Hospital Of CCUCM, Changchun 130021, China

## Abstract

**Purpose:**

Radiotherapy-induced sensorineural hearing loss (RISNHL) is a common adverse effect in patients with head and neck cancer. Given that there are few studies on the pathogenesis of RISNHL at present, we summarized the possible pathogenesis of RISNHL and possible protective measures found at present by referring to relevant literatures.

**Methods:**

We performed a comprehensive literature search in the PubMed database, using keywords “sensorineural hearing loss,” “radiotherapy,” and “cancer,” among others. The literature was examined for the possible mechanism and preventive measures of sensorineural hearing loss induced by radiotherapy.

**Results:**

We found that the incidence of RISNHL was closely related to the damage directly caused by ionizing radiation and the radiation-induced bystander effect. It also depends on the dose of radiation and the timing of chemotherapy. Studies confirmed that RISNHL is mainly involved in post-RT inflammatory response and changes in reactive oxygen species, mitogen-activated protein kinase, and p53 signaling pathways, leading to specific manners of cell death. We expect to reduce the incidence of hearing loss through advanced radiotherapy techniques, dose limitation of organs at risk, application of cell signaling inhibitors, use of antioxidants, induction of cochlear hair cell regeneration, and cochlear implantation.

**Conclusion:**

RISNHL is associated with radiation damage to DNA, oxidative stress, and inflammation of cochlear cells, stria vascularis endothelial cells, vascular endothelial cells, spiral ganglion neurons, and other supporting cells. At present, the occurrence mechanism of RISNHL has not been clearly illustrated, and further studies are needed to better understand the underlying mechanism, which is crucial to promote the formulation of better strategies and prevent the occurrence of RISNHL.

## 1. Introduction

Radiotherapy (RT) is commonly used and sometimes the first choice in treating head and neck tumors, especially nasopharyngeal carcinoma (NPC). Unfortunately, the complex anatomy and location of the tumor often lead to the exposure of the auditory pathway to ionizing radiation, which may result in conductive hearing loss (CHL) and sensorineural hearing loss (SNHL). SNHL is a tardive and irreversible complication that can be observed in patients with inner ear exposure to radiation fields. The incidence of SNHL varies widely, because of various reasons such as radiation dose, age, and hearing sensitivity of patients, ranging from 0 to 85% for low-frequency (<4 kHz) and from 27% to 95% for high-frequency (>4 kHz) SNHL. The affected hearing range usually starts with high frequencies and progresses toward lower frequencies [1].

Despite the increased interest in RT-induced ototoxicity in tumors of the head and neck tumors, there is little research on the mechanism and protection of radiotherapy-induced SNHL (RISNHL). This article is aimed at describing the progress made to understand the mechanism of RISNHL and the approaches developed so far for its protection or to reduce the associated complications.

## 2. Cochlear Structure and Function

**Figure 1:**
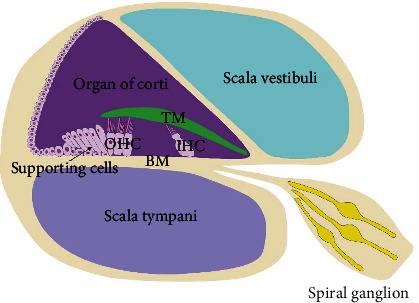
Cochlea structure diagram. BM: basement membrane; TM: tectorial membrane; IHC: inner hair cells; OHC: outer hair cells.

The impairment of cochlear structure has been shown to be associated with SNHL. A healthy neonatal baby's cochlea contains ~3500 inner hair cells (IHC) and 12,000 outer hair cells (OHC) (Figure 1) [2]. OHC comprises the basal and apical OHC that respond to higher and lower frequency sounds, respectively. Cochlear hair cells interlock with supporting cells to form an epithelial layer on top of the basilar membrane. Sound energy causes membrane motion and triggers K+ influx and hair cell (HC) receptor potential. Depolarization of OHCs forms the basis for cochlear sensitivity and frequency resolution, while depolarization of IHCs releases glutamate from ribbon synapses at their basal poles. Spiral ganglion neurons are bipolar neurons. When stimulated by IHCs, the bipolar spiral ganglion neurons innervating the IHCs transmit auditory information to the auditory center through extended synapses [3]. Cochlear hair cells (HCs) and spiral ganglion neurons constitute the basis of hearing function. These cells can hardly regenerate after death in mammals, making it increasingly important to protect these tissues during chemoradiotherapy.

## 3. Mechanism of RISNHL in Head and Neck Cancer

**Figure 2:**
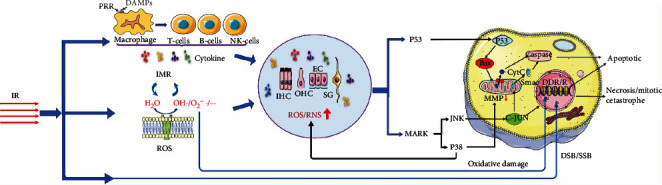
Mechanisms of cell damage induced by ionizing radiation (IR). Radiation can either damage DNA directly or cause oxidative damage to DNA through oxygen free radicals produced by ionizing water molecules, leading to cell death. Damage-associated molecular pattern molecules (DAMPs) will be released after cell damage or death, which activates macrophages and other antigen-presenting cells and enhances inflammatory and immune responses (IMR). Moreover, IMR and reactive oxygen species (ROS) can interact to change the cochlea microenvironment and induce cell death through p53 and mitogen-activated protein kinase (MAPK) signaling pathways. DDR/R: DNA damage response and repair reactions pathway; EC: endothelial cells; IHC: inner hair cells; OHC: outer hair cells; SG: spiral ganglion; MMP: mitochondrial membrane potential; DSB: double-strand breaks; SSB: single-strand breaks.

According to precious studies, ionizing radiation- (IR-) induced direct cell damage and radiation-induced bystander effect (RIBE) are responsible for hearing loss. Radiation exerts multiple effects by disrupting chemical bonds in all the basic components of the cells. These damages classified as direct and indirect damage to the deoxyribonucleic acid (DNA) could lead to multiple manners of cell fate decision, including apoptosis, autophagy, necrosis, and mitotic catastrophe [4]. The cell fate decision mainly depends on the type of cell and the severity of cell damage [5]. Furthermore, we describe the possible mechanism of cell damage by IR at the cellular and molecular levels (Figure 2).

### 3.1. Direct Effects of Radiation Induction

#### 3.1.1. Radiation-Induced DNA Damage

Damage is caused by IR primarily through local high-energy deposition in DNA [6] which causes single-strand breaks (SSB) and double-strand breaks (DSB) in DNA. After that, damages could be repaired [7]. DNA damage response and repair reaction pathways (DDR/R) mainly include single-strand break repair (SSBR), base excision repair (BER), and nucleotide excision repair (NER), which can initiate senescence or apoptosis when necessary. However, previous studies have confirmed that direct damage accounts for only about one-third of the biological effects of radiation. Most of the damage caused by radiation is mediated by free radicals generated during radiation exposure (which is classified as indirect damage), because most cells in the body are rich in water molecules [8, 9]. Increased ROS and NO after radiation have been proved to play a key role in the genotoxic effects of ionizing radiation [10].

#### 3.1.2. Radiation-Induced Formation of Reactive Oxygen Species and Signaling Pathway Activation

IR-induced DNA damage is mainly mediated by free radicals produced by the interaction between radiation and water molecules, which is classified as indirect damage [11]. Moreover, a consensus has been reached that reactive oxygen species (ROS) and reactive nitrogen species (RNS) in the cochlea often trigger caspase-mediated cell death after exposure to loud sound and ototoxic drugs [12, 13]. The excessive production of ROS and RNS induced by IR destroys the balance in the body and leads to oxidative stress. Oxidative stress can aggravate the inflammatory response, which in turn induces the production of ROS/RNS [14]. Oxidative active products such as ROS/RNS/nitric oxide (NO) can cause oxidative damage to DNA, and typical DNA oxidative damage leads to SSB and DSB [15, 16]. Meanwhile, a high level of NO/ROS leads to mitochondrial membrane leakage and causes functional defects. There is increasing evidence that oxidative stress products produced postradiation lead to the release of cytochrome C, decrease of mitochondrial membrane potential(MMP), and activation of p53, C-Jun N-terminal kinase (JNK), and mitogen-activated protein kinase (MAPK) in auditory cells [17–19]. At present, many studies have found that ROS can also lead to membrane lipid peroxidation, and the products of lipid peroxidation induced cell apoptosis through a variety of signaling pathways [20]. Overall, it can be inferred that ROS/RNS can lead to cell damage or death through various pathways.

MAPKs are intracellular proteins present in all eukaryotic cells and respond to extracellular and intracellular stimuli after phosphorylation. MAPKs intercept plasma membrane-bound receptor signals to activate transcription factors in the nucleus, collaborating with gene expression and facilitating the regulation of cell proliferation, differentiation, motility, and survival [21]. The JNK, also known as a pressure-activated protein kinase, is an important member of the MAPK family [22]. Furthermore, it has been shown that JNK is a major contributor to oxidative stress damage in mammalian inner ear cells after trauma. Inactive JNK located in the cytoplasm is activated upon exposure to environmental pressure and transported to the nucleus, consequently activating several transcription factors [23]. These transcription factors can regulate the cell stress response according to different influencing factors and lead to different outcomes (cell survival or death). Another way of JNK regulation is via its transportation to the mitochondria, which results in the release of the second mitochondria-derived activator of caspase (SMAC). SMACs can promote cell apoptosis by activating caspases [24]. The other important key mediator in the MAPK signaling pathway is the p38 family. Apoptosis mediated by p38 in HEI-OC1 cells was observed in a previous study [25]. The pharmacological inhibition of p38 before radiation exposure protects auditory cells in vitro from radiation by moderating changes in mitochondrial membrane potential (MMP) and generation of NO. Furthermore, p38 inhibitors protect neuroblasts from radiation-induced damage in vivo. These findings suggest that inhibition of p38 may be a plausible strategy for protecting the mitochondria from radiation.

The activation of p53 is known to induce cell cycle arrest, facilitating the conception of the apoptotic pathway. The proapoptotic member Bax is transcriptionally upregulated after p53 phosphorylation [12], prompting pore formation in the mitochondrial membrane. Activated nuclear p53 transports to the mitochondria, where it interacts with and inactivates prosurvival Bcl-2 proteins, simultaneously damaging the HCs directly [26]. Studies showed that phosphorylation of p53 protein increased dramatically at 3 h postirradiation. Several p53-regulated genes associated with cell cycle regulation and arrest were also found to increase, corresponding to the phosphorylation of p53 [27]. These findings suggest that the inhibition of p38/p53 activation could be a potential therapeutic strategy.

#### 3.1.3. Inflammatory Cell Recruitment

Radiation can induce inflammatory and immune response (IMR) and cause DNA damage [28]. Damaged cells release a variety of damage-associated molecular patterns (DAMPs), which activate antigen-presenting cells such as macrophages through pattern recognition receptor (PRR), causing IMR [29]. Several studies investigating the changes in immunomodulatory parameters after radiation have shown that these parameters maintain a chronic inflammatory state leading to radiation-related advanced pathology [28]. Furthermore, the continuous inflammatory process has been demonstrated as the principal cause of late IR effects [30]. After IR, the cells induce macrophages to produce various cytokines, such as IL-1, IL-6, IL-8, and TNF-*α*, causing cell damages [31]. Earlier studies have also reported an influx of inflammatory cells in the cochlea after acoustic trauma [32, 33]. Many studies have found that the stria vascularis plays a crucial role in the inflammatory process in the stria area by recruiting macrophages [34–36]. The role of this process in radiation-induced inflammatory response needs to be confirmed by future experiments. IR-induced IMR can alter the microenvironment of cochlear cells, leading to the occurrence of radiation-induced fibrosis [37, 38]. This could be one of the pathogeneses of RISNHL.

#### 3.1.4. Radiation-Induced Bystander Effect (RIBE)

RIBE has been extensively studied in recent years [6, 39, 40]. It was first proposed by Mole in the 1950s [41] and has been demonstrated in many studies, such as on breast cancer cells [42], human umbilical vein endothelial cells (HUVECs) [43], and lung cancer cell lines [44]. RIBE mainly includes DNA damage, malignant transformation, and cell death in bystander cells. The bystander cells are not exposed to radiation directly, but the asymmetry of DNA damage repair between the observer cells and the irradiated cells activates DDR/R of the observer cells and even induces their death.

**Figure 3:**
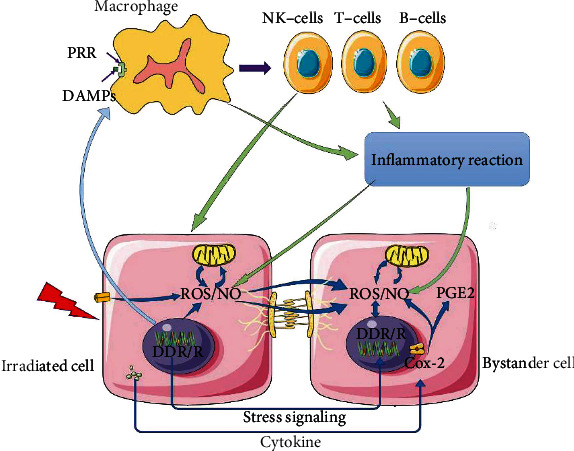
Radiation-induced bystander effect (RIBE). Ionizing radiation can directly cause DNA damage and activate the DNA damage response and repair reactions pathway (DDR/R), while the latter activates macrophages through the release of damage-associated molecular pattern molecules (DAMPs), thereby further enhancing the inflammatory and immune response. The reactive oxygen species (ROS)/nitric oxide (NO) and various cytokines produced by the damaged cells eventually crosstalk with the bystander cells through various paths, causing damage to the cells. PGE2: prostaglandin E2; PRR: pattern recognition receptor.

Oxidative stress also plays an important role in the RIBE. The main source of ROS causing bystander cell damage is the decomposition of irradiated cells [45]. At the same time, NADPH oxidase, bound to the plasma membrane of the observing cell, continuously produces ROS. Some studies have also shown that cytokines can stimulate COX-2 in bystander cells to release large amounts of ROS/NO during the production of prostaglandin E2, which promotes the transmission of RIBE [46]. RNS/NO is also produced by irradiated cells and are transmitted to bystander cells. The production of ROS/RNS/NO could lead to damage or death of bystander cells through a variety of pathways, similar to directly irradiated cells (Figure 3).

These results indicated that in the process of head and neck cancer radiotherapy, the cochlea cells could be damaged even without direct irradiation. Meanwhile, controlling the bystander effect with systemic/peripheral protection, rather than targeting the cochlea, may also reduce radiation damage. These all need to be explored.

### 3.2. Cell Death

Asenov et al. performed a histopathological examination of the temporal bone in patients with nasopharyngeal carcinoma (NPC) receiving chemotherapy and RT and revealed that denaturation of the stria vascularis, atrophy of the spiral ligament, reduction of spiral ganglion cells, and occasional loss of hair cells were the main changes in the cochlea [47].

#### 3.2.1. Cochlear HC Death

The loss of cochlear hair cells is primarily responsible for RISNHL [48]. The cochlea is reported sensitive to radiation [49]. Many studies have proved that free radical accumulation is an early event in HCs injury process [50, 51]. ROS have been found to originate from a wide range of sources, including direct radiation and bystander effects. For instance, an *in vitro* study showed that the fluorescence intensity of an oxidation-sensitive probe increased in a dose-dependent manner 1 h after the HC line OC-K3 was exposed to different doses of radiation (2/20/100 Gy) [27]. Furthermore, Sha et al. have speculated that the basal OHCs (respond to higher frequency sounds) could be more susceptible to free radical damage than the apical (respond to lower frequency sounds) OHCs due to a significantly lower level of the antioxidant-glutathione in the basal OHCs than the apical OHCs [52]. These results partially illustrated the underlying mechanism that RISNHL affects the high frequencies of the hearing range and progresses toward lower frequencies, which are consistent with clinical observations. And another study found that the probability of low-frequency SNHL after intensity-modulated radiation therapy (IMRT) treatment of NPC was 6–22%, and the probability of high-frequency SNHL was 37–51.2% [53]. It also indicates that the order of cell death caused by different sensitivity to ROS affects the degree of hearing loss.

What role do classic cellular signaling pathways play in this process? Previous studies have observed abnormal activation of JNK and P53 signaling pathways during the apoptosis of OHCs [54, 55]. The activation of the inducible transcription factor c-Jun is the core event in JNK-mediated apoptosis of oxidative stress-damaged auditory HCs [56]. However, several contradictory studies have also been reported questioning the role of JNK in hair cell apoptosis. For instance, a recent study showed that apoptosis occurred in a dose-dependent manner, mainly at 72 h postirradiation, whereas the early activation of c-JUN began at 3 h and then decreased at 24 h postirradiation, suggesting that the c-JUN pathway may not lead to radiation-induced apoptosis [27]. Therefore, further research is needed to elucidate the relationship between JNK, P53, and HC death after radiation.

#### 3.2.2. Stria Vascularis Endothelial Cell Death and Vascular Endothelial Cell Death

The stria vascularis is a specially stratified epithelial tissue of the outer wall of the cochlear duct with inner capillaries and is damaged after radiation exposure. These damages are not only caused by the direct effect of IR but also by various cytokines released by the irradiated cells. It has been proved that after irradiation, vascular endothelial cells in the stria vascularis was activated to produce aseptic inflammation [57, 58] which leads to thrombosis [59], endothelial cell contraction, and cell death. Additionally, Hellweg showed that radiation-induced genotoxicity is the main cause of aseptic inflammation, which activates the nuclear factor (NF)-*κ*B pathway [60]. Studies have also shown that the damage of the stria vascularis endothelial cells after high-dose irradiation may be related to the sphingolipid ceramide pathway [61], which is usually activated in epithelial cells after radiation-induced DNA damage. Reportedly, stria vascularis endothelial cell damage mainly affects the short- and medium-term hearing of patients after RT, especially in patients receiving single-fraction high-dose RT [14], whereas as evident from the histological results, stria vascularis cells were rarely damaged in fractionated RT. [62]

#### 3.2.3. Spiral Ganglion Neurons

It is well known that neurons are not capable of mitosis. However, owing to the damage of connective tissue cells (oligodendrocytes, astrocytes) involved in myelination and other supporting functions, nerve tissue is radiosensitive as a whole [63].

Though spiral ganglion cells were greatly affected by radiation during fractional RT [62], their function impairments after RT are controversial. In a study of NPC patients who underwent radiotherapy alone, no significant difference was found in evoked response audiometry and pure-tone threshold audiometry(PTA) before and after radiotherapy [64].

However, another study showed that after RT, patients' PTA increased significantly. The latency of auditory brainstem response (ABR) waves was prolonged or even disappeared at 1 year after RT, the amplitude of I-IV waves was reduced, and the latency between the peaks of I-V waves was significantly prolonged [65]. The experimental results of Lau et al. are also consistent with the above experiments [66]. Previous studies have revealed that the first wave (I) of ABR reflects the activity of the spiral ganglion cells [67]. The sign of neuroinflammation is the occurrence of radiation-induced fibrosis. Its pathological process includes axonal injury and demyelination, as well as the proliferation of fibrotic tissue around the nerve trunk and the decrease of vascular supply [37]. Krysta et al. found that the density of spiral ganglion cell bodies and surrounding protrusions exposed to 60 Gy IR was significantly decreased in mice, and the related extracellular matrix was also lost. In the 20 Gy IR group, only a slight threshold shift, with pathological examination revealing signs of vacuolation and separation of spiral ganglion cells, and early demyelination [68]. While demyelination is also found responsible for the hearing loss caused by excessive sound stimulation [69] and aging [70]. These findings indicate that inhibition of the demyelination of spiral ganglion neurons can help alleviate hearing loss.

## 4. Protection Measures

Radiation-related severe late complications, such as hearing loss, negatively affect the quality of life. Therefore, the prevention and treatment of SNHL have also attracted attention. Different strategies have been tested at the clinical and preclinical levels to reduce the incidence of RT induced hearing impairment. From hearing loss to cell death-induced deafness, we can reduce the occurrence of sensorineural hearing loss from many aspects. These include improved radiotherapy technology, limitation of normal tissue dose, application of signaling inhibitors, use of antioxidants, induction of cochlear hair cell regeneration, and cochlear implant.

### 4.1. Improvement of Radiotherapy and Chemotherapy Regiments

The degree of hearing loss increases with the dose of the inner ear [71, 72]. The most important measure to prevent RISNHL is to reduce the radiation dose in the cochlea [73]. A recent study found that the effect of fractionated stereotactic radiotherapy (fSRT) on the total dose of cochlea may be less than that of stereotactic radiosurgery (SRS) [74]. Studies have also shown that NPC patients have an increased risk of developing high-frequency SNHL when the radiation dose to internal auditory canal (IAC) was IAC − D_max_ > 42.13 Gy or IAC − D_mean_ > 32.71 Gy [75]. Hence, reducing the cochlear dose seems to be critical to reducing the incidence of SNHL [73]. Furthermore, the dose distribution of organs at risk (OARs) is related to the T stage, especially tumor volume (GTV) [76, 77]. It has been demonstrated that the cochlear radiation dose can be reduced depending on different T stages, using the technique of dose-limiting stratification scheme, for example, by setting the limit dose threshold of 45 Gy or lower for T1 and T2 stages or by slightly adjusting the angle of the specific beam radiated to the target [78]. A comparative study of Smartarc-based volumetric-modulated arc therapy (VMAT-S) and stepped field intensity-modulated radiotherapy (IMRT) treatment of locally advanced NPC found that the average cochlear dose of the VMAT-S regimen was lower than that of the IMRT regimen [79].

Previous studies have found that the incidence of SNHL increases when chemoradiotherapy (CRT) is used. One of the studies found that the incidence of SNHL was 84% in CRT group, but only 26% in RT group [80, 81]. But for many advanced tumors, we usually need a combination of radiotherapy and chemotherapy. Many clinical studies have proved the superiority of CRT [82]. Platinum-based chemotherapy regimens have been shown to have definite ototoxic side effects. The molecular mechanisms of cisplatin-induced ototoxicity include the imbalance of endogenous oxidation system and antioxidant system, cochlear inflammatory response, and abnormal activation or inactivation of P53, HSP, CDK2, etc. [83]. A comparative study indicated a higher incidence of ototoxicity in patients receiving cisplatin after RT than in patients receiving chemotherapy before RT. [84] It may be associated with increased exposure to cisplatin in the Organ of Corti or auditory nerve after RT. It has been shown that radiation induces increased vesicle transport, phosphatase activity, and endothelial cell tight junction opening, which increase the permeability of the blood–brain barrier in a dose-dependent manner. The increased permeability increases the exposure of the Organ of Corti or auditory nerve to cisplatin. This explains the increased incidence of SNHL after concurrent cisplatin administration or RT. [85] In the application of platinum-based chemotherapy, drugs that reduce ototoxicity can also be used prophylactically, including drugs targeting endogenous antioxidant system (allopurinol, Ebselen, NOX3/RNS inhibitor, curcumin, ferulic acid), drugs targeting cochlear inflammation (TNF-*α* neutralizer, capsaicin, EGCG), and drugs targeting p53/HSP/CDK2. Drug-coated nanoparticles can also be used to increase the blood drug concentration in the cochlea, so as to reduce the ototoxicity of chemotherapeutic drugs [83, 86].

### 4.2. Signal Pathway Inhibitors

Cell damage and death are the result of cell signaling pathway transduction. It may be possible to save irradiated cells by inhibiting part of the signaling pathway. Previous studies have investigated the role of JNK inhibitors and P38 inhibitors in hearing protection. AM-111, a JNK inhibitor developed by Auris Medical, has been shown to protect hearing in animals and is currently undergoing clinical trials to study the treatment of sudden deafness [87]. Considering that RISNHL is also involved in JNK signaling pathway, it is expected to be used in RISNHL. P38 mitogen-activated protein kinase inhibitors such as SB203580 have been shown to prevent RISNHL in both cell and animal studies [25].

### 4.3. Antioxidant Drugs

The occurrence of RISNHL is closely related to oxidative stress. Many antioxidant drugs have been studied to treat various types of hearing loss. Studies have shown that mitochondrial targeted antioxidants are far superior to nontargeted cellular antioxidants in reducing mitochondrial oxidative damage [88]. Nontargeted antioxidants have been shown to protect ototoxicity in many cell experiments and animal experiments. This paper introduces the application of several antioxidants in hearing loss (Table 1), such as melatonin, amifostine, L-carnitine, methylprednisolone, piracetam, and epicatechin [89–94]. These drugs have not yet been tested in clinical trials to prove their feasibility in humans, and further clinical studies are expected. The concentration of traditional antioxidants in mitochondria is very low. Mitochondrial-targeted antioxidants discovered in recent years can accumulate in mitochondria and effectively inhibit oxidative stress response. MitoQ and SkQR1 are two kinds of mitochondrial targeted oxidants, which have been proved to have protective effects against ototoxicity in many studies [95, 96].

### 4.4. Cochlea HC Regeneration

Cochlear HCs rarely regenerate. However, in recent years, it has been proposed that HCs can be regenerated by Atoh1 gene transfer. In this study, adenovirus containing Atoh1 gene was injected into the cochlea of deaf guinea pigs. Eight weeks after injection, different degrees of hair cell regeneration were observed, and hearing was significantly improved. “CGF166”—an adenovirus vector encoding the human unregulated transcription factor “Hath1”—is currently in clinical trials [87]. If proven safe and effective, this could be a major milestone in hearing loss treatment.

### 4.5. Cochlear Implants

Cochlear implants allow sound waves to bypass damaged HCs and travel to the brain. Hybrid cochlear implants are designed for adults with high-frequency SNHL [97]. Cochlear implantation is undoubtedly a good method for patients with SNHL whose HCs are damaged but the afferent nerve pathway is not damaged. A case-control comparative study indicated that RISNHL was mainly due to the damage of the cochlea structure, and the function of the auditory nerve was basically preserved [98]. However, there is a contrary suggestion that there is damage to the retrocochlear auditory pathway following radiotherapy, including demyelination and neuroinflammation as mentioned above [65]. More complete clinical studies are needed to verify whether the retrocochlear auditory pathway is damaged after radiotherapy.

## 5. Conclusions

Currently, the possible mechanisms of RISNHL can be simplified as cells exposed to radiation lead to cell death through direct or indirect DNA damage, inflammatory cell recruitment, ROS-mediated oxidative stress response, and activation of multiple signaling pathways (such as MAPK and P53). Cells that are not exposed to radiation die in various forms as a result of a series of oxidative stress and inflammatory responses released or triggered by damaged cells. Meanwhile, ROS generated by ionizing H_2_O do harm to DNA or mitochondrial membrane through oxidative stress reaction and inflammatory reaction. Sensorineural hearing loss is closely related to the damage of hair cells, stria vascularis, vascular endothelial cells, and spiral ganglion. Histopathological examination of patients after radiotherapy and chemotherapy showed loss of hair cells, striatal degeneration, atrophy of spiral ligaments, and reduction of spiral ganglion cells. The selection of radiation dose and timing of combined chemotherapy are key factors for the occurrence of RISNHL.

The most important way to prevent RISNHL is to reduce radiation damage to the hearing system through dose stratification with precise radiotherapy techniques. In view of various molecular mechanisms of cell death, signal pathway inhibitors, antioxidants, hair cell regeneration, and cochlear implant can be used to prevent or alleviate hearing loss after radiation. Various protective measures known to date are the result of in vitro evaluations or animal studies. Further clinical trials are needed to confirm their protective effect.

## Figures and Tables

**Table 1:** Studies on the protective effect of antioxidants on various SNHL.

Drug	Experimental design	Outcome	Mechanism	Reference
MT	Animal experiment: rats	DPOAE: M+RT group and RT+M group>RT group	Inhibit production of ROS7; enhance the DNA repair process	[89]
AMF	Animal experiment: guinea pigs	Degree of cochlear hair cell damage: IRR + AMF group < RT group. No difference between 100 mg/kg and 200 mg/kg AMF groups	Be hydrolyzed into active component *in vivo*, which has sulfhydryl group that scavenges free radicals	[90]
LC	Animal experiment: guinea pigs	Histopathological examination: LC can ameliorate radiation-induced cochlear damage in guinea pigs.	Improving mitochondrial oxygen utilization and scavenging free-radicals	[91]
MP	Clinical trials: NPC patients	Pure tone audiometry; DPOAE; ABR: the use of MP during RT can reduce the early RISNHL	Mechanisms to protect the OHC: 1. Affects transcription and inhibits caspase-mediated apoptosis 2. Inhibits inflammatory response 3. Promotes GSH synthesis	[92]
PIR	Animal experiment: male albino guinea pigs	Histopathologic examination: PIR might reduce radiation-induced cochlear damage in guinea pigs	Increases oxygenation in the tumor cells with its rheological effects and decreases apoptosis in surrounding healthy cells	[93]
EC	Cell experiment: HEI-OC1 and UB-OC1 Animal experiment: embryos/rats	EC can increase the survival rate of HEI-OC1 cells after radiotherapy. EC can inhibit the ABR threshold in the rats.	Inhibits ROS production and MAPK activity	[94]

MT: melatonin; AMF: amifostine; LC: L-carnitine; MP: methylprednisolone; PIR: piracetam; EC: epicatechin; GSH: glutathione.

## Data Availability

All data, models, and code generated or used during the study appear in the submitted article.
